# Detection and characterization of polioviruses originating from urban sewage in Yaounde and Douala, Cameroon 2016–2017

**DOI:** 10.1186/s13104-019-4280-6

**Published:** 2019-05-02

**Authors:** Daniel Kamga Njile, Serge Alain Sadeuh-Mba, Marie-Claire Endegue-Zanga, Marcellin Nimpa Mengouo, Marlise Dontsop Djoumetio, Franky Baonga Ba Pouth, Ousmane Madiagne Diop, Richard Njouom

**Affiliations:** 1 Virology Service, National Reference and Public Health Laboratory, Centre Pasteur of Cameroon, 451 Rue 2005, PO box 1274, Yaounde, Cameroon; 2 World Health Organization, Country Office, PO box 155, Yaounde, Cameroon; 3 Expanded Program on Immunization, Ministry of Public Health, Yaounde, Cameroon; 4 The Polio Eradication Department, World Health Organization, Avenue Appia 20, 1211 Geneva 27, Switzerland

**Keywords:** Poliovirus, Vaccine, Surveillance, Eradication, Sewage, Cameroon

## Abstract

**Objective:**

Transmission of wild polioviruses (WPVs) and vaccine-derived polioviruses (VDPVs) have been interrupted in Cameroon since July 2014. Subsequently, Cameroon withdrew Sabin type 2 from routine immunization in April 2016. This study aimed to investigate the detection rates and overtime distribution of the types of PVs recovered from urban sewage in Cameroon.

**Results:**

From January 2016 to December 2017, 517 sewage specimens originating from Yaounde (325 specimens) and Douala (192 specimens) were analyzed. No WPVs and VDPVs were isolated in this study. In contrast, vaccine strains of poliovirus were detected throughout the study period. Isolates Sabin types 1 and 3 were sporadically detected whereas Sabin 2 was found only from January to May 2016 both in Yaounde and Douala. The absence of Sabin 2 in sewage specimens since June 2016 indicates its rapid disappearance after withdrawal from routine immunization in April 2016. This study provides substantial support to the observation that WPV and VDPVs have been successfully eliminated in Cameroon. However, it remains essential to maintain and extend high quality environmental surveillance as long as WPV reservoirs and VDPV outbreaks are detected in Africa.

**Electronic supplementary material:**

The online version of this article (10.1186/s13104-019-4280-6) contains supplementary material, which is available to authorized users.

## Introduction

Enteroviruses (EVs) are ubiquitous human pathogens belonging to the genus *Enterovirus* and the family *Picornaviridae*. The genus *Enterovirus* include Non-Polio EVs (NPEVs) that infect both humans and animals as well as Polioviruses (PVs) that specifically infect humans [1, 2]. There is three serotypes of PVs (PV1, PV2, PV3) and each type is further divided into three categories based on the extent of nucleotide sequence divergence of their VP1 capsid coding gene compared to that of corresponding oral poliovirus vaccine (OPV) strains: (i) OPV-like or Sabin viruses (< 1% divergent for types 1 and 3, and < 0.6% for type 2), (ii) Vaccine-derived PVs [VDPVs] (1–15% divergent for PV1 and PV3 and 0, 6–15% divergent for PV2), and (iii) Wild PVs [WPVs] (> 15% divergent) [3].

Originally, the strategy of the Global Polio Eradication Initiative in endemic countries mainly relied on the assessment of PV circulation through Acute Flaccid Paralysis (AFP) surveillance among children < 15 years and extensive immunization with live-attenuated OPV. This strategy has led to the reduction of the incidence of WPV-associated poliomyelitis from an estimated 350,000 cases in 1988 to only 22 cases in 2017: 14 in Afghanistan and 8 in Pakistan [4]. At the same time, circulating VDPVs were reported only in two countries in 2017: 74 in Syria and 22 in Democratic Republic of Congo [5, 6].

PVs are transmitted by the feco-oral route through contaminated food, water and objects [7]. After ingestion, PV particles multiplies in the oropharynx and the intestine, and is excreted in stools for 4–6 weeks. The risk of PV infection and transmission has been shown to be correlated with poor hygiene and sanitary conditions as well as high population density [8–10]. Most PV infections are generally asymptomatic but they can induce poliomyelitis in less than 1% of infected cases. Therefore, efficient AFP surveillance target only 1% of infected cases. Therefore the detection of PVs in environment samples is a useful supplement to monitor PV circulation in communities in absence of clinical presentations on which AFP surveillance relies.

Indigenous WPV transmission has originally been interrupted in Cameroon in 1999. However, WPV types 1 and 3 were repeatedly imported from endemic reservoirs into Cameroon from 2003 to 2014 [11]. In particular, isolation of highly evolved lineage of WPV type 1 associated to the 2013–2014 outbreak suggested potential gaps in the AFP surveillance system and vaccine coverage in Cameroon [11]. Moreover, circulating VDPVs, genetically linked to a previous outbreak in the neighboring Chad, caused a poliomyelitis outbreak in the Extreme Nord region of Cameroon in 2013 [12]. Appropriate responses with supplementary immunization activities were successful in stopping WPV and VDPV in Cameroon as from July 2014. Since July 2014, no clinical case of WPV and VDPV infections have been reported in Cameroon where national estimates of PV vaccine coverage ≥ 83% have been documented from 2014 to 2017 [13]. In April 2016, Cameroon switched from trivalent OPV (tOPV; Sabin types 1, 2, and 3) to bivalent OPV (bOPV; Sabin types 1 and 3) after the introduction of one dose of inactivated PV vaccine type 2 in the routine immunization schedule. This switch was accompanied with the setup of environmental surveillance of PVs since January 2016.

This study aimed to (i) confirm the absence of circulating WPVs and VDPVs and (ii) investigate the detection rates and overtime distribution of the types of PVs recovered from urban sewage in Cameroon.

## Main text

### Methods

Between January 2016 and December 2017, 12 wastewater collection sites, including 8 in Yaounde and 4 in Douala, were selected on sewage drains covering populations at risk of PV transmission in Yaounde and Douala (Table 1). Collection sites were selected where wastewater flows were available from sites characterized by poor sanitation and high population density (Additional file 1: Figure S1). One liter of wastewater specimen was collected twice a week at each site on the due day. Specimens were transported in a reverse cold chain (4–8 °C) to the Reference Laboratory for Poliomyelitis surveillance at the Centre Pasteur of Cameroon. Upon arrival, the pH of the specimens were eventually adjusted between 7 and 7.4 before virus concentration by the two-phase separation method using Dextran T40 and polyethylene glycol 6000 (PEG 6000) according to the standard protocol [14].

**Table 1:** Number of virus types detected in the resulting isolates from environmental surveillance conducted in Yaounde and Douala between January 2016 and December 2017

Towns and sampling sites	2016													
	SL1	SL1 + SL2	SL2	SL2 + SL3	SL3	NPEV + SL1	NPEV + SL1 + SL2	NPEV + SL1 + SL3	NPEV + SL2	NPEV + SL3	NPEV	Negative samples	Positive samples	All samples
Yaounde														
Aurore											3	*23*	*3*	*26*
Maetur Mendong		1		1							6	*10*	*8*	*18*
Melen Elobi					1						3	*11*	*4*	*15*
Mokolo Market										1	4	*18*	*5*	*23*
Mvog-Ada	1		1								9	*14*	*11*	*25*
Nkolndongo												*11*		*11*
Nkomkana				1		1	1		1		8	*12*	*12*	*24*
Sports Palace											8	*18*	*8*	*26*
Total Yaounde	*1*	*1*	*1*	*2*	*1*	*1*	*1*		*1*	*1*	*41*	*117*	*51*	*168*
Douala														
Cité des Palmiers	1		2					1			5	*16*	*9*	*25*
Derrière Jet Hotel					1						3	*10*	*4*	*14*
Pamplemousse Drain									1		9	*15*	*10*	*25*
Camp Yabassi Bridge				1	1	1			1		5	*15*	*9*	*25*
Total Douala	*1*		*2*	*1*	*2*	*1*		*1*	*2*		*22*	*56*	*32*	*89*
Total general	*2*	*1*	*3*	3	*3*	*2*	*1*	*1*	*3*	*1*	*63*	*173*	*83*	*257*

Towns and sampling sites	2017								2016–2017		
	SL1	SL3	NPEV + SL1	NPEV + SL3	NPEV	Negative samples	Positive samples	All samples	Positive samples	All samples	Positive samples (%)
Yaounde											
Aurore						*2*	*0*	*2*	*3*	*28*	*10.7*
Maetur Mendong					5	*12*	*5*	*17*	*13*	*35*	*37.1*
Melen Elobi	1				3	*17*	*4*	*21*	*8*	*36*	*22.2*
Mokolo Market					8	*15*	*8*	*23*	*13*	*46*	*28.3*
Mvog-Ada		1		1	8	*14*	*10*	*24*	*21*	*49*	*42.9*
Nkolndongo		1	1		7	*16*	*9*	*25*	*9*	*36*	*25.0*
Nkomkana	2	2			5	*11*	*9*	*20*	*21*	*44*	*47.7*
Sports Palace		1		2	10	*12*	*13*	*25*	*21*	*51*	*41.2*
Total Yaounde	*3*	*5*	*1*	*3*	*46*	*99*	*58*	*157*	*109*	*325*	*33.5*
Douala											
Cité des Palmiers				1	6	*17*	*7*	*24*	*16*	*49*	*32.7*
Derrière Jet Hotel					7	*17*	*7*	*24*	*11*	*38*	*28.9*
Pamplemousse Drain				2	7	*17*	*9*	*26*	*19*	*51*	*37.3*
Camp Yabassi Bridge		1		1	7	*20*	*9*	*29*	*18*	*54*	*33.3*
Total Douala		*1*		*4*	*27*	*71*	*32*	*103*	*64*	*192*	*33.3*
Total general	*3*	*6*	*1*	*7*	*73*	*170*	*90*	*260*	*173*	*517*	*33.5*

Mixtures of two or more viruses are specified with the “+” sign linking them

For clarity, values equal to zero (0) have been omitted; except those in the raws corresponding to sub-totals and totals that are highlighted in italic

SL1: Type 1 Sabin-like poliovirus; SL2: type 2 Sabin-like poliovirus; SL3: type 3 Sabin-like poliovirus; NPEV: non-polio enterovirus

Water concentrates were analyzed according to the World Health Organization (WHO) guidelines for environmental surveillance of PV circulation [14] and the WHO polio laboratory manual [15]. These analyses comprised virus isolation, molecular differentiation of PV isolates, and sequencing of the VP1 capsid coding gene of Sabin 2 and other PV isolates identified as non-vaccine by molecular differentiation.

Two cell lines were used in this study: (i) L20B which are mouse fibroblast cells that have been transfected to express the PV-specific receptor CD155 and (ii) RD which are human rhabdomyosarcoma tumor cells expressing the majority of EV receptors including those for PVs. A volume of 500 μL of each wastewater concentrate was inoculated into 5 flasks of L20B and 5 flasks of RD cells cultures maintained in Eagle’s Minimum Essential Medium (Sigma-Aldrich) with 2% decomplemented fetal calf serum at 36 °C. Inoculated flasks were observed under an inverted objective microscope for 5 consecutive days to search for cytopathic effects (CPE). Isolates showing CPE only on RD but not on L20B cell cultures were classified as NPEVs. Those showing CPE on L20B cells cultures were considered as PVs.

Suspected PV isolates were typed by Intratypic differentiation (ITD) using real time RT-PCR (rRT-PCR) amplification with a combination of oligonucleotide sets as previously described [15, 16]. This assay is able to identify the type of PV isolate and to discriminate between their wild and vaccine-related strains. Since type 2 OPV has been withdrawn from routine immunization, all Sabin 2 isolates identified by ITD were confirmed by the sequencing of their full-length VP1 capsid coding gene [17].

### Results

A total of 517 sewage samples (325 from Yaounde and 192 from Douala) were collected from January 2016 to December 2017. EVs were detected in 33.5% (173/517) of samples: 33.5% (109/325) in Yaounde and 33.3% (64/192) in Douala (Table 1, Additional file 1: Figure S1). Isolates were obtained from all studied sites in Douala in 2016 and 2017 whereas two sites in Yaounde (Nkoldongo and Aurore) showed no culture positive specimen in 2016 and 2017, respectively (Table 1). Remarkably, the Aurore site showed a virus isolation rate as low as 10.7% (3/28) while the isolation rates from other sites ranged from 22.2 to 47.7% and were comparable among the sites in both cities (Table 1). Both NPEV and PV detection rates were comparable between Yaounde and Douala (P ≥ 0.2).

**Fig. 1:**
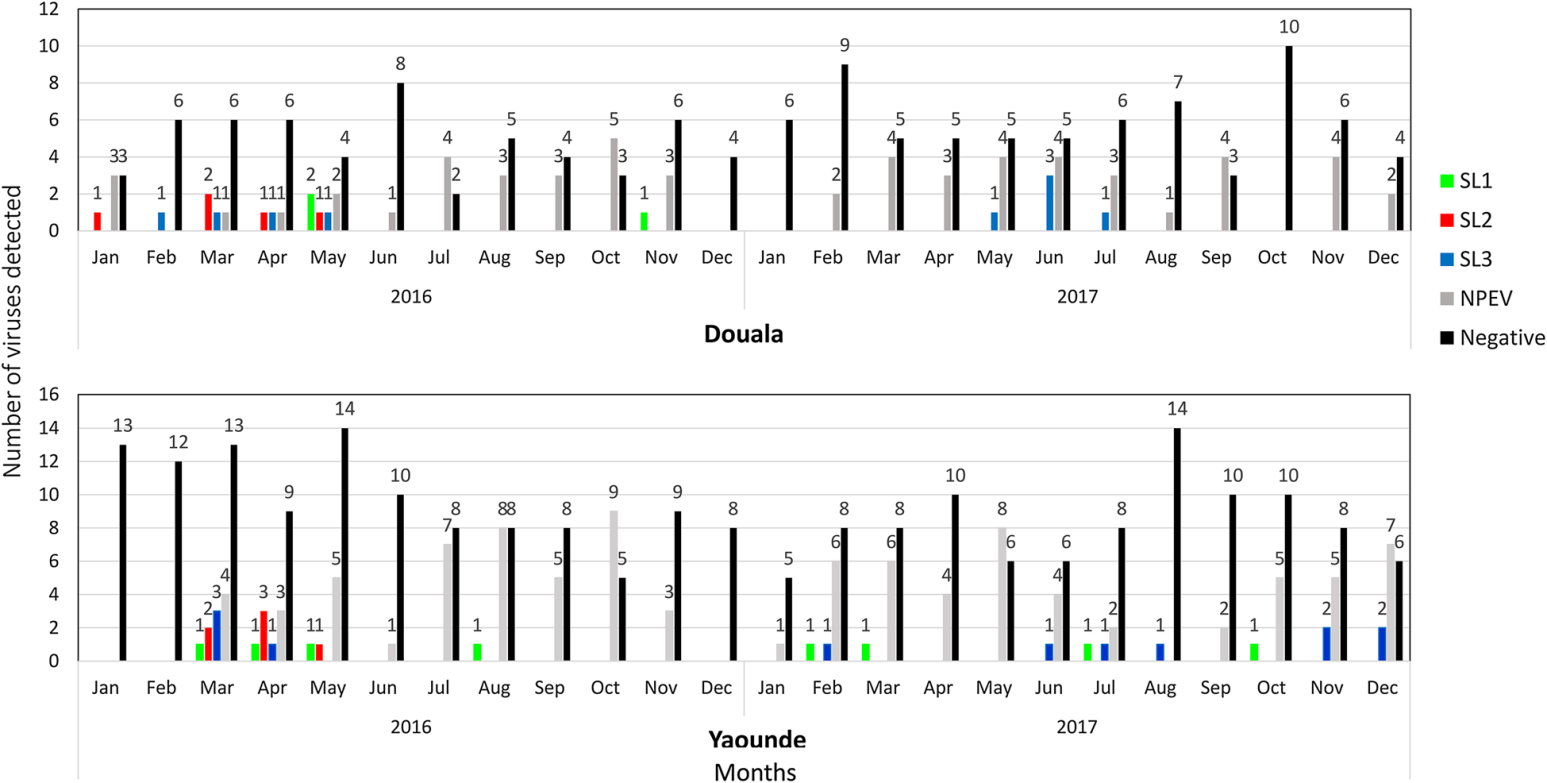
Temporal pattern of the isolation of non-polio enteroviruses and vaccine polioviruses in the cities of Yaounde and Douala from January 2016 to December 2017. The number of individual virus types (SL1: Sabin type 1; SL2: Sabin type 2; SL3: Sabin type 3; NPEV: non-polio enterovirus) isolated and culture negative samples are indicated for each months

As expected, NPEVs represented the highest proportion of viruses detected irrespective of sample origin and month of collection (Fig. 1). NPEVs were detected in 136 (26.3%) of samples including 87 (26.7%) in Yaounde and 49 (25.5%) in Douala. In contrast to NPEVs, PVs were detected only in 37 (7.2%) sewage samples including 22 (6.8%) in Yaounde and 15 (7.8%) in Douala (Table 1).

Multiple virus types were simultaneously detected from some sample (Table 1). Considering individual isolates, a total of 43 PV isolates (26 from Yaounde and 17 from Douala) were recovered from the 37 L20B-positive samples (Table 2). In contrast to NPEV that were consistently detected in all months in Yaounde and Douala during the study period, Sabin isolates were sporadically detected with comparable rates between Yaounde and Douala (P ≥ 0.8).

**Table2:** Number of samples analyzed and viruses detected in isolates resulting from environmental surveillance conducted in Yaounde and Douala between January 2016 and December 2017

Cities and sampling sites	2016						2017						Total 2016–2017	
	n	SL1	SL2	SL3	NPEV	All PVs	n	SL1	SL2	SL3	NPEV	All PVs	n	All PVs (%)
Yaounde														
Aurore	26				3	0	2					0	28	0 (0.0)
Maetur Mendong	18	1	2	1	6	4	17				5	0	35	4 (11.4)
Melen Elobi	15			1	3	1	21	1			3	1	36	2 (5.6)
Mokolo Market	23			1	5	1	23				8	0	46	1 (2.2)
Mvog-Ada	25	1	1		9	2	24			2	9	2	49	4 (8.2)
Nkolndongo	11					0	25	1		1	8	2	36	2 (5.6)
Nkomkana	24	2	3	1	11	6	20	2		2	5	4	44	10 (22.7)
Sports Palace	26				8	0	25			3	12	3	51	3 (5.9)
Total Yaounde	*168*	*4*	*6*	*4*	*45*	*14*	*157*	*4*	*0*	*8*	*50*	*12*	*325*	*26 (8.0)*
Douala														
Cité des Palmiers	25	2	2	1	6	5	24			1	7	1	49	6 (12.2)
Behind Jet Hotel	14			1	3	1	24				7	0	38	1 (2.6)
Pamplemousse Drain	25		1		10	1	26			2	9	2	51	3 (5.9)
Camp Yabassi Bridge	25	1	2	2	7	5	29			2	8	2	54	7 (13.0)
Total Douala	*89*	*3*	*5*	*4*	*26*	*12*	*103*	*0*	*0*	*5*	*31*	*5*	*192*	*17 (8.9)*
Total general	*257*	*7*	*11*	*8*	*71*	*26*	*260*	*4*	*0*	*13*	*81*	*17*	*517*	*43 (8.3)*

For clarity, values equal to zero (0) have been omitted; except those in the raws corresponding to sub-totals and totals that are highlighted in italic

Although a pattern of seasonality could not be ruled out in this study, differences in the overtime distribution were noticeable in both cities. Sabin types 1 and 3 were isolated throughout the study period but their temporal distribution were apparently more dispersed in Yaounde than Douala (Fig. 1). Sabin type 2 displayed the same temporal distribution in both cities and their detection were limited between January and May 2016. Analysis of the VP1 sequence of these Sabin 2 isolates showed ≤ 2 nucleotide difference compared to the original OPV type 2.

Interestingly, neither WPV nor VDPV were detected throughout the study period. These findings provide a substantial support to the observation from AFP surveillance which indicates that WPV and VDPV transmission has been successfully interrupted in Cameroon since 2014.

### Discussion

The continuous isolation of NPEVs from sewage specimens from January 2016 to December 2017 confirms the extensive circulation of NPEVs as previously found among several populations in Cameroon [18–20]. The sporadic isolation of Sabin types 1 and 3 throughout the study period is consistent with the fact that Sabin 1 and Sabin 3 are still being used in routine immunization in Cameroon. In accordance with resent updates on the progress towards PV eradication [4, 21], no WPV isolate was identified during this study in Cameroon, thus suggesting the absence of silent WPV transmission in Yaoundé and Douala between January 2016 and December 2017.

Since the certification of the eradication of WPV type 2 in 2015, all countries that were using OPV switched from tOPV to bOPV from mid-April to mid-May 2016 [22]. Since then, the number of Sabin 2 isolated from both AFP cases and environmental specimens have progressively decline worldwide [23]. Accordingly, this study identified Sabin 2 isolates only during the first semester of 2016 (Fig. 1). The absence of WPV, VDPV as well as the early disappearance of Sabin 2, 1 month after the switch from tOPV to bOPV, are likely due to the high vaccine coverage of the target population in Cameroon. Indeed, national estimates of PV vaccine coverage was ≥ 83% between 2014 and 2017 in Cameroon compared to Nigeria where they have steadily been at 40% during the same period [13]. Transmission of WPV type 1 was originally thought to have also been interrupted in the neighboring Nigeria since July 2014 [24]. However, prolonged transmission of undetected WPV type 1 was recently reported in the Borno State of Nigeria [25]. Since 2016, type 2 circulating VDPV associated outbreaks have also been recently documented in multiple locations including Democratic Republic of Congo, Nigeria, Somalia, Pakistan and Syria; likely as result of the reduction of the population immunity against the PV type 2 [5, 24, 26, 27]. Despite the absence of WPVs and circulating VDPVs in Cameroon since 2014, it remains essential to maintain high quality AFP surveillance supplemented by extended environmental surveillance in order to ensure rapid detection and control of VDPV emergence and WPV importations in polio-free countries.

## Limitations

Although the present study is the first to investigate potential silent circulation of vaccine PVs, cVDPVs and WPVs in Cameroon, we focused only on the two biggest cities of Cameroon that are Yaounde and Douala. It would have been more interesting to include regions of Cameroon that share borders with the northern states of Nigeria that are still considered as endemic for PVs. This limitation is already being addressed through the ongoing extension of environmental surveillance of PVs in Cameroon.

## Additional file

**Additional file 1: Figure S1.** Map of Douala and Yaounde indicating selected sampling sites where sewage specimens were collected for environmental surveillance for enteroviruses. The cities of Yaounde and Douala are indicated by black circles on the map of Cameroon.

## Abbreviations

AFP: Acute Flaccid Paralysis
CPE: cytopathic effects
EVs: enteroviruses
ITD: intratypic differentiation
NPEV: non polio enterovirus
OPV: oral poliovirus vaccine
PV: poliovirus
PV1, 2, 3: poliovirus types 1, 2, 3
VDPV: vaccine-derived poliovirus
WPV: wild poliovirus
WHO: World Health Organization
